# Green Synthesis of Silver Nanoparticles and Polymeric Nanofiber Composites: Fabrications, Mechanisms, and Applications

**DOI:** 10.3390/polym17172327

**Published:** 2025-08-28

**Authors:** Hany M. Abdelmoneim, Tarek H. Taha, Abdulrahman Mohammed Alhudhaibi, Feras M. Afifi, Abdullah A. Faqihi, Sulaiman A. Alsalamah, Hamdi Bendif

**Affiliations:** 1 Microbiology Department, Faculty of Science, Ain Shams University, Cairo 11566, Egypt; hanymoneim2010@gmail.com; 2 Department of Biology, College of Science, Imam Mohammad Ibn Saud Islamic University (IMSIU), Riyadh 11623, Saudi Arabia; amalhudhaibi@imamu.edu.sa (A.M.A.); saalsalamah@imamu.edu.sa (S.A.A.); hlbendif@imamu.edu.sa (H.B.); 3 Department of Physics, Al-Leith University College, Umm Al-Qura University, Makkah 24382, Saudi Arabia; 4 Department of Industrial Engineering, College of Engineering and Computer Science, Jazan University, P. O. Box 706, Jazan 45142, Saudi Arabia; afaqihi@jazanu.edu.sa

**Keywords:** green synthesis of silver nanoparticles, polymeric nanofibers, antimicrobial activity, wound dressing, food packaging, wastewater treatment

## Abstract

This manuscript reviews the green synthesis of silver nanoparticles (AgNPs) and their incorporation into polymeric nanofiber composites. It discusses various synthesis methods, emphasizing eco-friendly biological approaches over chemical and physical ones due to their cost-effectiveness and reduced toxicity. The review emphasizes the enhanced antimicrobial properties of AgNPs and their composites, particularly in electrospun nanofibers, for diverse biomedical, environmental, and industrial applications. It also covers the characterization, properties, and mechanisms of AgNPs, along with the advantages of combining them with polymers such as PVA and PEO, as well as cyclodextrin, to create novel functional nanocomposites.

## 1. Introduction

Nanotechnology is the science of the manipulation of matter on a very small scale, with a size in the range of 1–100 nm [1,2,3]. Nanotechnology is one of the most vital fields of research in materials science, focusing on the synthesis and applications of nanoparticles [4]. There has been considerable interest in synthesizing composite materials at the nanoscale, at which materials have unique chemical, physical, optical, magnetic, and electrical properties, which leads to many potential applications [4,5,6]. Composite nanomaterials in which noble metal nanoparticles are dispersed in a polymer matrix may display novel physical and chemical properties of great scientific and technological importance [7]. Metal nanoparticles are important due to their potential applications in catalysis, photonics, biomedicine, antimicrobial activity, and optics [8]. Among all the noble metals, silver has been used as an antimicrobial agent since ancient times, with historical records showing its use by ancient Egyptian, Greek, Roman, and Phoenician civilizations for water purification, wound treatment, prevention of infections, and food preservation [9,10]. It has gained much attention due to its medicinal, clinical, and culinary properties [11,12].

Silver nanoparticles (AgNPs) can be synthesized using various methods, such as chemical, physical, and biological methods [13,14]. However, the chemical and physical methods are costly, quite complicated, and potentially harmful to the environment due to the toxic chemical compounds used as reducing agents [15,16]. The biological approaches, also known as “green synthesis methods,” have recently received more attention due to their ability to avoid the use of toxic chemicals as well as being cost-effective and eco-friendly [17,18]. Recent studies have highlighted the effectiveness of plant-mediated green synthesis of AgNPs, emphasizing their enhanced antimicrobial properties and potential applications in biomedicine and environmental remediation [19,20]. Therefore, both the research and industrial sectors are currently interested in the biosynthesis of AgNPs for use in many biomedical, environmental, and industrial applications [21]. It is also widely used for therapeutic purpose due to its antibacterial, antifungal, anti-inflammatory, antiviral, and anticancer activities [22,23,24,25,26]. Recent proteomic and mechanistic studies have revealed their broad-spectrum biocidal potential and synergy with antibiotics, further strengthening their biomedical relevance [27]. Numerous microorganisms, such as fungi, bacteria, yeast, and actinomycetes, which can produce AgNPs through intracellular or extracellular pathways [4]. Among them, bacteria have received the most attention in the biosynthesis of nanoparticles due to their rapid growth, ease of handling, and genetic modification [28,29]. Extracellular production is preferred because it eliminates the need for complex extraction and purification steps [30,31].

Recent advances in nanomaterials science have highlighted the potential of engineered nanomaterials for a variety of applications. For instance, the surface engineering of M5X4 MXenes is being explored for next-generation energy solutions, focusing on how modifying their surface chemistry can enhance their electronic, electrochemical, and optical properties [32]. This approach of tuning material properties at the nanoscale is conceptually similar to the green synthesis of AgNPs and their integration into polymeric nanofiber composites. AgNPs have a limited range of applications when used alone, but when incorporated into the polymer matrix, their applicability is greatly enhanced [33]. The combination of nanofibers and nanoparticles technologies maximizes their structural properties, making them an ideal mixture for many applications [34]. Polymeric nanofiber composites containing AgNPs have been reported to exhibit significant antimicrobial activity against pathogenic bacteria [35,36]. Nanofibrous materials consist of fibers with a diameter less than 500 nm, and have several advantages over their bulk counterparts [37]. The properties of nanofibers, such as nanometric thickness, controllable porosity, and high contact surface area, enable potential applications in various fields [34]. Furthermore, nanofibers have the ability to combine with nanoparticles either in their bulk or on their surface to create so-called “nanofibrous composite materials” [38]. Various techniques, such as phase separation, self-assembly, and electrospinning, have been proposed for the synthesis of nanofibrous materials [39]. Of the various fabrication methods, the electrospinning stands out for its low cost, versatility, and single-step setup [40]. Recent advancements have demonstrated the successful incorporation of AgNPs into electrospun nanofibers, enhancing their antimicrobial efficacy and potential for biomedical applications [6].

Natural and synthetic polymers are increasingly combined to produce composite nanofibers. Polyvinyl alcohol (PVA) and polyethylene oxide (PEO) are hydrophilic polymers with excellent biocompatibility, mechanical performance, and low toxicity [36]. They are also easily electrospinnable due to their high molecular weight and viscosity [41]. Beta-cyclodextrins (β-CDs), although not polymers, have attractive properties when incorporated into electrospun fibers. These cyclic oligosaccharides form inclusion complexes with a wide range of molecules that alter the apparent solubility and stability of the guest molecule [42]. When incorporated into a polymer matrix, they can help control the release rate [43]. The addition of β-CD in polymer nanofibers would be extremely attractive since the composites have novel characteristics, which can potentially improve the application areas of nanofibers [7,44]. In green synthesis, polymer-based nanoparticles serve as both scaffolds for coordinating silver ions (Ag^+^) and as electron donors to facilitate reduction of Ag^+^ to elemental silver (Ag^0^). The functional groups present on the polymer, such as amines, phenols, and carbonyls, play a crucial role in determining the speed of the reduction process (reduction kinetics) and the stability of the resulting colloidal nanoparticles [45,46]. Chitosan and lignin utilize different chemical mechanisms for reduction reactions. Chitosan, with its high amine density, forms transient Schiff bases to reduce Ag^+^ [47], while lignin employs redox-active phenolics for the same purpose [48]. This behavior contrasts with synthetic polymers like PVP, where terminal hydroxyls drive reduction [49]. For instance, polyphenols facilitate single-electron transfers that result in the formation of semiquinone radicals [50]. In contrast, polysaccharides are involved in two-electron transfers that proceed through aldehyde intermediates [51]. The ultimate morphology of the nanoparticles is further determined by the stabilization mechanism, which can be either steric, as observed with polyvinylpyrrolidone (PVP) [52], or electrostatic, as seen with chitosan [53].

In light of these developments, the specific objective of this review is to provide a focused, up-to-date synthesis of (i) green synthesis strategies of AgNPs, (ii) their antimicrobial applications, and (iii) their integration into polymeric nanofiber composites, with particular attention to fabrication techniques, functional mechanisms, application potential, and future challenges. This targeted perspective aims to guide interdisciplinary research linking nanotechnology, microbiology, and materials science toward practical applications in healthcare, food safety, and environmental protection.

In brief, this review is discussing the approach of green synthesis, characterization, properties, and applications of AgNPs. It also covers the processing, characterization, and properties of AgNP-containing polymeric nanofibers, followed by their considerable antimicrobial activity and applications.

## 2. Nanotechnology

Nanotechnology is the field of applied science that deals with matter at the molecular and atomic scales, usually 100 nm or smaller, and it focuses on this range when used in materials or devices [54,55]. Due to its ability to control particle size to fit functions that would otherwise be impossible to fit using conventional methods, nanotechnology has seen rapid growth in the last few decades [40,56]. It is an emerging cutting-edge technology in many different scientific fields, including biology, chemistry, and materials science [13]. This includes polymer science, polymer-based biomaterials, nanoparticles for drug delivery, miniemulsion particles, layer-by-layer self-assembled polymer films, electrospun nanofibers, imprint lithography, polymer blends, and nanocomposites [57]. The novel classes of nano-based materials have received significant attention due to their essential advantages, including bioimaging, biocompatibility, functionalization and tumor targeting [58]. Nanomaterials typically range in size from 1 to 100 nm. This size is about 10^−9^, or a billionth of a meter [27,54]. On the other hand, the developing simple, reliable, green, and eco-friendly procedures for the synthesizing nanomaterials is an important aspect of nanotechnology [59]. Biogenic nanotechnology constitutes an area of interest with the important objective of facilitating the production of nanomaterials, such as metal nanoparticles [60]. Several studies have reported that metal nanoparticles could be incorporated into polymeric nanofibers to impart antimicrobial activity for biomedical applications [61]. Among these metal nanoparticles, AgNPs have been widely used as a bioactive polymeric material due to their catalytic activity, antimicrobial activity, higher conductivity, and non-toxic nature [62,63]. Therefore, AgNPs represent potent candidates for the developing innovative and effective nanotechnology-derived biocompatible nanostructured materials for unconventional antimicrobial applications [64,65,66].

## 3. Nanoparticles (NPs)

Nanoparticles are small fragments with a nanoscale diameter that ranges between 1–100 nm, with very good thermal conductivity, catalytic reactivity, non-linear optical performance, and chemical stability due to their large surface area-to-volume ratio [67,68]. They are generally classified into different groups based on sizes, shapes, and properties. The different groups include carbon-based nanoparticles, metal nanoparticles, ceramic nanoparticles, polymeric nanoparticles, and many others [69,70,71]. Metal nanoparticles, as inorganic nanoparticles, exhibit very different physical and chemical properties than conventional bulk metal [72]. The properties of nanoparticles depend on their size and shape, as well as their composition [73]. They have a high surface area-to-volume ratio, which confers unique properties on them and enhances their catalytic, magnetic, mechanical, and optical properties, thereby expanding their potential biomedical applications [74,75,76]. Nanoparticles can be synthesized using multiple methods, including chemical, physical, and biological ones [27]. Silver nanoparticles are considered to be one of the most used nanoparticles by researchers due to their unique properties such as high conductivity, chemical resistance, antibacterial, antiviral, antifungal, anti-angiogenic, and anti-inflammatory properties in different fields of medicine [68,72]. The incorporation of AgNPs into polymeric nanofibers has attracted considerable attention due to their strong and broad-spectrum antimicrobial activity [77], sensitive and specific detection ability of DNA [78], plasmonic effect as catalysts [79], and superior electrical conductivity [80,81].

## 4. Silver Nanoparticles (AgNPs)

### 4.1. Silver, Background Information

Silver is a shining white metallic element, placed 47th in the periodic chart with Ag, meaning “Argentum”, as its chemical symbol [82]. Silver is one of the fundamental elements that make up our planet. It is a rare but naturally occurring element that is slightly harder than gold, and very ductile and malleable [83,84]. Pure silver has the highest electrical and thermal conductivity of all metals and has the lowest contact resistance [85]. Silver can be found in four different oxidation states: Ag^0^, Ag^+^, Ag^2+^, and Ag^3+^. The first two are the most abundant; the last two are unstable in the aquatic environment. The free silver ion is Ag^+^ [83,86]. Silver has been widely used for thousands of years in human history for applications including jewelry, utensils, monetary currency, dental alloy, photography, and explosives [87]. Among silver’s many applications, those exploiting its disinfectant property for hygienic and medicinal purposes are time-honoured and prominent. Hippocrates, the father of modern medicine, believed that silver powder had beneficial healing and anti-disease properties, and it was considered an ulcer treatment [82]. Silver as an ion was used in earlier civilizations, particularly in Egypt. This silver ion was used mainly in wound dressings to treat wounds that were hard to heal [88]. However, advancements in modern science have helped silver regain its lost luster. Metallic silver is subjected to new engineering technologies that result in extraordinary novel morphologies and characteristics. Instead of being made “large,” metallic silver is transformed into ultrafine particles whose size is measured in nanometers (nm). When these particles have at least one dimension less than 100 nm, they are called nanoparticles [71,89].

### 4.2. Characteristics

AgNPs have been widely explored among various metallic and non-metallic nanoparticles due to their applicability and versatility [90,91,92]. AgNPs are nanoscale structures made of silver atoms that have been metallically bonded together. AgNPs are a group of silver atoms ranging in size from 1 to 100 nm, which can be synthesized with various approaches using different precursors, reductants, and capping agents [26,93]. Physical and chemical properties of AgNPs—including surface chemistry, size, size distribution, shape, particle morphology, particle composition, coating/capping, agglomeration, dissolution rate, particle reactivity in solution, efficiency of ion release, cell type, and finally, type of reducing agents used for synthesis—are crucial factors for determination of cytotoxicity of AgNPs [94]. At the nanoscale, particles exhibit different physical, optical, and chemical properties owing to the dominance of quantum mechanics [93,95]. AgNPs have among the best antimicrobial characteristics covering a wide range of pathogenic microorganisms [68]. They exhibit very strong bactericidal activity against both Gram-positive and Gram-negative bacteria, including multi-resistant strains [76,96]. Also, AgNPs have received wide attention in wastewater treatment, biomedicine, drug delivery, vector control, and agriculture [68,97]. The efficient conductivity of AgNPs has increased their applications in a wide array of products, such as electronic devices, inks, adhesives, and pastes, and in controlling microbial growth and infections, which has also made them eco-friendly [98,99]. AgNPs have drawn tremendous academic and industrial interest due to their antibacterial, fungicidal, and antiviral properties [36,68,100,101,102,103,104,105,106,107].

### 4.3. Methods of AgNP Synthesis

**Figure 1:**
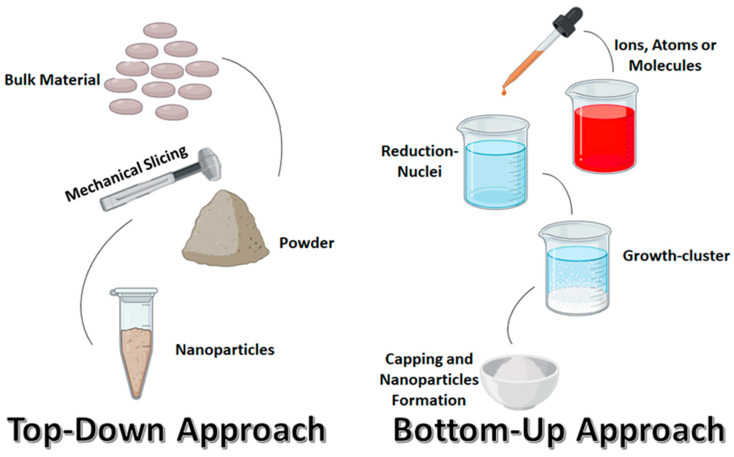
An overview of top-down and bottom-up methods of nanoparticle synthesis. Adopted from [108].

AgNPs are synthesized using various physical, chemical, and biological methods, which results in different shapes and sizes for use in numerous applications [13,76]. These methods of synthesis are categorized into two main categories, namely “top-down” and “bottom-up” approaches (Figure 1) [20,71,108]. In the top-down approach, the size of silver metal in its bulk form is reduced mechanically to the nanoscale, while the bottom-up approach (self-assembly) involves the dissolution of silver salts in a solvent, reducing silver ions to their element with the use of a reducing agent, and then stabilizing the resulting neutral silver nanoparticles with stabilizing agents to prevent agglomeration [14,109,110].

#### 4.3.1. Top-Down Approach

In this synthesis method, a destructive approach is used to produce AgNPs. It begins with breaking down a larger molecule, which is decomposed into smaller units, and then these units are converted into suitable nanoparticles [26,110]. It is energy-demanding and requires extensive processing. The major advantage of this technique is the control of the size distribution and morphologies of nanoparticles [111,112]. This approach, also known as physical synthesis methods, includes laser ablation, lithography, grinding or milling, spray pyrolysis, evaporation–condensation, and other decomposition processes [13,68].

#### 4.3.2. Bottom-Up Approach

The bottom-up technique is the reverse of the top-down method. It involves bringing together atoms and molecules to produce a diverse range of nanoparticles [26,110]; therefore, this approach is also called the “building-up approach.” The sol–gel process, laser pyrolysis, aerosol process, chemical vapor deposition, and biological agent-assisted synthesis are a few examples of this approach [68,113]. In this approach, nanoparticles can be synthesized using either chemical or biological methods by the self-assembly phenomenon of atoms into new nuclei, which grow into nanoscaled particles [112,114]. The green and biological method of bottom-up synthesis of nanoparticles has recently attracted more attention from many researchers. In green synthesis of nanoparticles there is no need to use high temperature, pressure, or energy, or toxic chemicals [13,112,115]. In this approach, AgNPs are synthesized using biological systems, including various sources such as bacteria, fungi, algae, and plant extracts [20,116].

## 5. Biosynthesis of AgNPs Using Plant Extracts

The bioactive molecules found in plants and their parts include polysaccharides, fats, proteins, amino acids, nucleic acids, pigments, enzymes, vitamins, and several types of secondary metabolites, which act as reducing agents to produce nanoparticles from metal salts without producing any toxic by-products [112,117,118]. Several studies have been carried out on the synthesis of silver nanoparticles using plant extracts. Gardea-Torresdey et al. [119] were the first to report the synthesis of AgNPs using alfalfa sprouts. Ahmad and Sharma, Ref. [15] also synthesized AgNPs using *Ananas comosus* extract and characterized the synthesized AgNPs using different techniques. Velmurugan et al. [120] also reported the synthesis of AgNPs using peanut shell extract. In other studies, AgNPs were also synthesized using bark extract of *Saraca asoca* [121], leaf extract of *Boswellia sacra* [16], and banana peel extract [122]. Similarly, *Vitis vinifera* [123], Andean blackberry [124], *Adansonia digitata* [125], *Nitraria schoberi* [126], or multiple fruit peels have also been reported for AgNPs synthesis [127]. Combinations of plant extracts have also been reported [128]. Furthermore, different parts of different plants, e.g., peel, seed, fruit, bark, flower, stem, and root, are also used in the nanoformulations of AgNPs [2,68].

## 6. Biosynthesis of AgNPs Using Microorganisms

**Figure 2:**
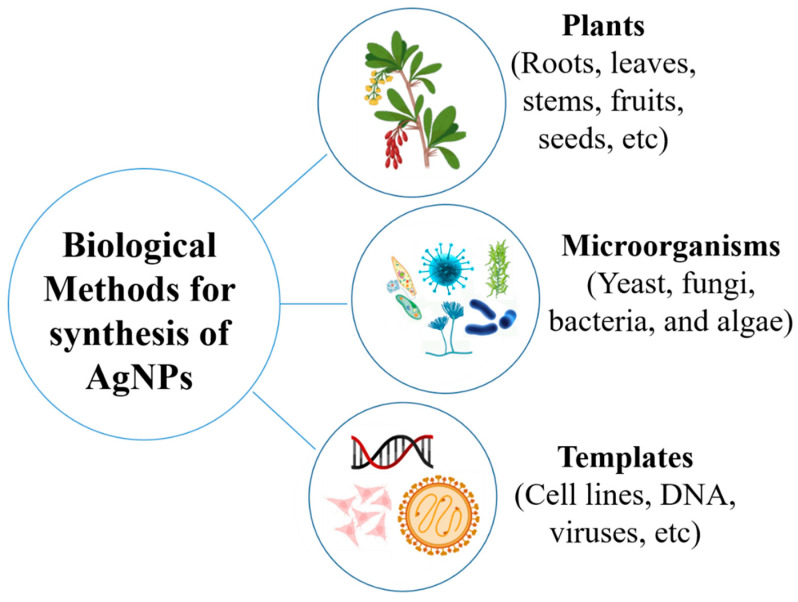
Various approaches to the synthesis of AgNPs. Adopted from [2].

Among the various biological sources for the green synthesis of metallic nanoparticles, the green synthesis mediated by microorganisms has acquired a special place due to their high growth rate, ease of cultivation, and the ability to grow in ambient conditions of temperature, pH, and pressure [129]. Microorganisms such as bacteria, fungi, and algae are of great interest in the synthesis of nanoparticles [68], although this process faces challenges such as cultural contamination, lengthy procedures, and less control over the size of nanoparticles (Figure 2) [2,130]. The synthesis of nanoparticles can be carried out both extracellularly and intracellularly using microbes [31,131]. Not all microorganisms are capable of producing metallic nanoparticles because they are produced by metabolic pathways and through cellular enzymes that may not be present in certain organisms [132].

### 6.1. Biosynthesis of AgNPs Using Bacteria

Diverse groups of bacteria inhabit the soil, water, plants, and animals. They can survive under various soil pH, salinity, temperature, and nutrient conditions. Some of these can occur in highly contaminated or hyper accumulated soils and plants [129]. The bacteria are known to possess the extraordinary ability to reduce heavy metal ions and are therefore considered one of the best candidates for the synthesis of AgNPs [13,20,68]. In the case of bacteria, the first synthesis of AgNPs was established using the strain *Pseudomonas stutzeri* AG259 that was isolated from a silver mine [133]. Several studies have reported the synthesis of AgNPs using bacteria [13]. According to studies, some bacterial species, such as *Pseudomonas stutzeri* and *Pseudomonas aeruginosa*, are developing the ability to use specific defense mechanisms to overcome stresses like the toxicity of heavy metal ions or metals, and even survive and grow in the presence of high metal ion concentrations [134]. Klaus et al. [135] reported the synthesis of AgNPs with well-defined compositions and shapes using *Pseudomonas stutzeri* strain. This study was among the earliest studies that synthesized AgNPs using bacteria. Kalimuthu et al. [136] synthesized AgNPs using the biomass of the bacterium *Bacillus licheniformis*. Extracellular production is more prioritized than intracellular, which requires extraction and purification of AgNPs from the microbial growth [30,31,68]. In addition, the extracellular production was confirmed to include high amounts of proteins, which acted as capping agents [137]. Saravanan et al. [138] reported the extracellular biosynthesis of highly stable AgNPs using the culture supernatant of *Bacillus megaterium* NCIM 2326. Similarly, Nour El-Dein et al. [30] reported that the crude metabolite of *Escherichia coli* D8 (MF062579) was able to synthesize AgNPs within 1–2 min using a green and cost-effective method. Moreover, some important bacterial species that can contribute to the biosynthesis of AgNPs through intracellular and extracellular mechanisms are *Escherichia coli*, *Klebsiella pneumoniae*, *Actinobacter* sp., *Bacillus cereus*, *Lactobacillus* spp., and *Pseudomonas aeruginosa* [139]. A study by Shahverdi et al. [140] found the rapid biosynthesis of metallic silver nanoparticles by using the culture supernatants of Enterobacteriaceae strains. This is the first report focusing on the biosynthesis of AgNPs by using culture supernatants of different Enterobacteriaceae strains, including *Klebsiella pneumoniae*, *Escherichia coli*, and *Enterobacter cloacae.* Abdelmoneim et al. [31] were the first to report the synthesis of AgNPs using the culture supernatant of *Leclercia adecarboxylata*.

### 6.2. Biosynthesis of AgNPs Using Fungi

Fungi are another group of microorganisms that can be used as excellent sources of a wide variety of enzymes for the synthesis of different metallic nanoparticles, including AgNPs [26,141]. The synthesis of AgNPs by fungi has attracted more attention due to their economical large-scale production with controlled size and low residue toxicity [2,112,142]. The mechanism of synthesizing of nanoparticles using fungi could be intracellular or extracellular [13,112]. It has been reported that silver ions are reduced extracellularly in the presence of fungi to produce stable AgNPs in water [20,143]. Kathiresan et al. [144] reported the synthesis of AgNPs using the culture supernatants of *Penicillium fellutanum* isolated from coastal mangrove sediment. Jain et al. [145] synthesized AgNPs by extracellular synthesis methods using cell-free filtrate of *Aspergillus flavus* NJP08. A study by Ma et al. [146] reported the extracellular biosynthesis of AgNPs using the cell-free filtrate of the fungal strain *Penicillium aculeatum* Su1 as a reducing and stabilizing agent. Similarly, Lotfy et al. [147] reported that the mycelial filtrate of *Aspergillus terreus* BA6 was used to reduce AgNO_3_, which acts as an effective biofactory for the biosynthesis of AgNPs.

### 6.3. Biosynthesis of AgNPs Using Algae

Aquatic microorganisms, specifically algae, have been used and reported to synthesize AgNPs [26]. They range in size from microscopic (picoplankton) to macroscopic (Rhodophyta) [2]. Microalgae can be used as an efficient bionanofactory, able to produce metallic nanoparticles by reducing various metal ions such as silver, gold, and cadmium [148,149]. Both the live and dried biomass of microalgae can be used to synthesize metallic nanoparticles. Xie et al. [150] reported the biosynthesis of AgNPs using the extracted biomolecules of the unicellular green microalgae *Chlorella vulgaris*. In another study reported by Merin et al. [151], AgNPs were synthesized using the microalgae *Chaetoceros calcitrans*, *Chlorella salina*, *Isochrysis galbana*, and *Tetraselmis gracilis*. Prasad et al. [152] reported for the first time the synthesis of AgNPs using the extracts of the brown marine algae *Cystophora moniliformis* as a reducing and stabilizing agent. The biosynthesis of AgNPs was carried out extracellularly using a marine cyanobacterium, *Oscillatoriawillei* NTDM01, which reduced silver ions and stabilized the produced AgNPs with a secreted protein [129]. Several microalgae such as *Chlorella vulgaris*, *Spirulina platensis*, and *Lyngbya majuscule* have been utilized for biosynthesis of AgNPs [149,153].

### 6.4. Biosynthesis of AgNPs Using Miscellaneous Sources

Recently, the use of DNA as a reducing agent has been driven by nanotechnology [2,14,76,154]. The high affinity of DNA bases for silver makes it a model stabilizer [155,156]. Kasyanenko et al. [157] reported that AgNPs were synthesized on DNA strands and found to be possibly located at the N^7^ guanine and phosphate. Another attempt was made with the DNA of the calf thymus to synthesize AgNPs [156,158].

Interest in the synthesis of nanomaterials by using a virus as the aid template is rising. The virus-mediated production of nanoparticles has been studied by researchers. Plant and animal viruses show a capacity for nanoparticle synthesis, but animal viruses are not studied very much as plant viruses due to safety concerns [159]. In addition, the various byproducts of plant metabolism can be utilized for the fabrication of nanoparticles, such as resins, gums, and biowaste of sugar crystallization “molasses” [159].

Several approaches highlighting the variety of natural sources utilized and the resulting characteristics of the AgNPs were summarized to provide a comparative overview of recent advancements in the green synthesis of AgNPs (Table 1).

### 6.5. Mechanism of Biosynthesis of AgNPs

**Figure 3:**
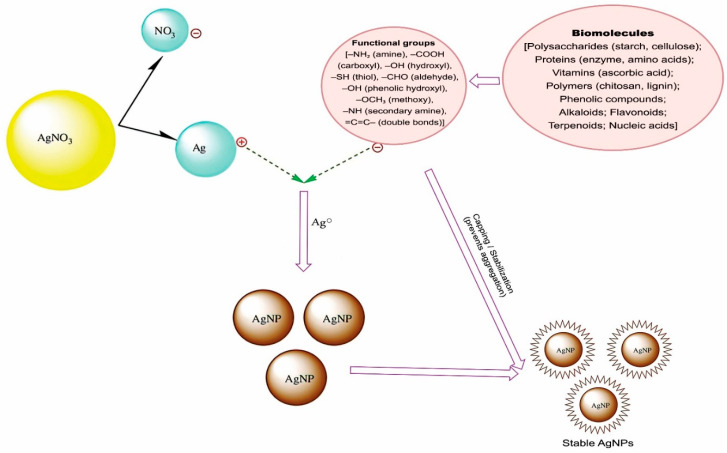
Schematic representation illustrating the reduction of Ag^+^ ions to stable Ag^0^ nanoparticles by different biomolecules.

The synthesis of AgNPs by biological entities is due to the presence of a large number of organic chemicals such as carbohydrates, fats, proteins, enzymes, coenzymes, phenols, flavonoids, terpenoids, alkaloids, gum, etc., which are able to donate electrons for the reduction of Ag^+^ ions to Ag^0^ (Figure 3) [26]. The active ingredient that reduces of Ag^+^ ions varies depending on the organism or extract used. For the nanotransformation of AgNPs, electrons are thought to be derived from the dehydrogenation of acids (ascorbic acid) and alcohols (catechol) in hydrophytes, the conversions of keto to enol in mesophytes, or both mechanisms in xerophytes plants [179,180]. The microbial cellular and extracellular oxidoreductase enzymes can perform similar reduction processes [180,181].

## 7. Factors Affecting the Synthesis of AgNPs

Optimization of the factors affecting the characteristics of AgNPs is crucial for a more efficient and controllable synthesis process [36,147]. The main physical and chemical parameters influencing the synthesis of AgNPs are the reaction time, concentration of metal ions, reaction temperature, extract content, pH of the reaction mixture, substrate concentration, and agitation [26,181]. Parameters such as the reaction duration, the concentration of metallic ions, and the extract composition have a significant effect on the shape, morphology, and size of the AgNPs [14,182]. Salem and Fouda, Ref. [183] reported that the optimum concentrations of metal ions, temperature, and pH of the reaction mixture play a key role in the synthesis of nanoparticles. The pH of the reaction mixture has a significant effect on the growth of AgNPs because it changes the electrical charges of the biomolecules, probably affecting their reducing and capping properties [184,185,186]. Most researchers have reported the relevance of neutral or basic pH for the synthesis of AgNPs due to the better stability of the synthesized nanoparticles in basic medium [187,188,189,190]. Some other advantages mentioned regarding basic pH are rapid growth rate, good yield, monodispersity, and enhanced reduction process [14,181,191,192,193,194]. At low pH, aggregation occurs during nucleation, leading to the formation of large nanoparticles, whereas high pH has produced very stable small nanoparticles [26,186,195]. Small and uniform nanoparticles were synthesized by increasing the pH of the reaction mixture [194,196,197]. On the other hand, some researchers have indicated that an acidic pH is more favorable than an alkaline pH for the formation of AgNPs [198]. In this regard, Ibrahim [122] found that an acidic pH (4.5) was more appropriate for the synthesis of AgNPs using banana peel extract. Verma and Mehata [199] reported that absorption intensity was highest at a pH of 13, but caused instability and aggregation of AgNPs when kept overnight. The effect of pH on the synthesis of AgNPs could be also attributed to the availability of the reducing agents. It has been recorded that abundant reducing agent at elevated pH facilitates the reduction of silver ions into AgNPs, while the reduced concentration of the reducing agents at lower pH slows the silver ions reduction process [200].

The reaction conditions, such as time of stirring and reaction temperature, are also significant parameters. Many researchers used temperatures up to 100 °C for the synthesis of AgNPs using biopolymers and plant extracts, while the use of mesophilic microorganisms restricted the reaction temperature to 40 °C [181]. At higher temperatures, the mesophilic microorganisms die as a result of inactivating their vital enzymes. The increase in temperature (30 to 90 °C) resulted in an increase in the rate of the synthesis of AgNPs [68,201] and also favored the synthesis of smaller-sized AgNPs [202]. In general, most researchers synthesized AgNPs at room temperature (25 to 37 °C) [181,198]. We could attribute the change of the AgNP products at different temperatures to the effect of the applied temperature on the action of the reducing agent. It has been proved that the reaction of the reducing agent is accelerated at elevated temperatures resulting in variable rates of nanoparticles’ nucleation and growth [203].

Reaction time is also essential parameter that affects the synthesis of AgNPs [68]. The color change to brownish yellow and a peak SPR in the range 400–500 nm indicated the initiation of the formation of nanoparticles. In order to ensure the complete consumption of silver ions, reaction time plays an important role [198]. In general, the intensity of the absorbance peak increases with time as the number of nanoparticles increases [204,205]. The size, shape, and extent of the synthesis of AgNPs are strongly dependent on the concentration of silver ions [68]. The concentration of silver metal salt most commonly used was found at 1.0 mM [198]. However, other concentrations of 0.01, 0.1, 0.5, 1.75, 2, 3, 5, 7, 8, 10, 20, 58.8, and 100 mM were also reported [198,206,207,208]. The absorption peak increases as the concentration of silver ions increases [31,209].

## 8. Characterization of AgNPs

The physicochemical properties of nanoparticles are important for their behavior, biological distribution, safety, and efficacy [94]. The special properties of AgNPs will determine their potential and application [68,210]. The shape, size, morphology, and surface area of the synthesized metallic nanoparticles are evaluated using various characterization techniques. These techniques include ultraviolet–visible spectrophotometry (UV-Vis), transmission electron microscopy (TEM), high-resolution transmission electron microscopy (HRTEM), scanning electron microscopy (SEM), field emission scanning electron microscopy (FESEM), Fourier transform infrared spectroscopy (FTIR), X-ray diffraction (XRD), atomic field microscopy (AFM), zeta potential (ZP), dynamic light scattering (DLS), and selected area electron diffraction (SAED) [20,109,153]. Some important characterization techniques for evaluating AgNPs include UV-Vis, SEM, TEM, FTIR, and XRD [19,118,181]. The appearance of a yellow to slightly brownish-yellow color in the colorless solution was taken as an indication of the synthesis of AgNPs by almost all the researchers [181,211]. AgNPs have unique optical properties that make them strongly interact with specific wavelengths of light [94,212]. The SPR peak of the synthesized AgNPs was observed in the range of 400–450 nm, the significant range for AgNPs [213]. UV-vis spectroscopy is a very useful and reliable technique for the primary characterization of synthesized nanoparticles, which is used to monitor the synthesis and stability of AgNPs [94,214]. UV-vis spectroscopy is used as a characterization technique immediately after synthesizing AgNPs of size from 2 to 100 nm in the range wavelength of 300–800 nm [109,212,215]. TEM is a valuable, frequently used, and important technique for characterizing of nanomaterials, used to obtain quantitative measurements of particle and/or grain size, size distribution, and morphology [19,94,216,217]. SEM is a technique that uses electrons rather than light to form an output image [118]. SEM is a surface imaging technique fully capable of resolving different particle sizes, size distributions, nanomaterial shapes, and the surface morphology of the synthesized particles at the micro- and nanoscales [212,216,218,219]. Modern high-resolution SEM is capable of identifying the morphology of nanoparticles below 10 nm [94,212]. FTIR spectroscopy is frequently used to determine the possible functional groups of the biomolecules responsible for the reduction, capping, and efficient stabilization of AgNPs [211,220] and the local molecular environment of the capping agents on the nanoparticles [118]. FTIR dissects functional groups in various absorbance regions between 4000 and 400 cm^−1^ [221,222]. XRD is a useful tool for obtaining information about the atomic structure of materials. X-ray diffraction is one of the most extensive conventional methods for characterizing AgNPs. The crystallographic structure and morphology, which comprise the crystalline structure, lattice parameters, crystalline size, and the nature of the phase, are determined using the XRD technique [20,210]. X-rays are electromagnetic radiation, similar to light. However, X-rays have a much shorter wavelength compared to light. They are created when particles with electrical charges decelerate [221]. A comprehensive characterization is essential to confirm the synthesis, stability, and functional properties of AgNPs. Table 2 summarizes the key analytical techniques employed for this purpose, detailing the information each method provides.

A comparative table with most of the available parameters such as the synthesis method, particle size, polydispersity, zeta potential, and scalability challenges are represented (Table 3).

## 9. Stability, Aggregation, and Shelf Life of AgNPs

The stability and long-term efficacy of AgNPs are critically dependent on preventing their aggregation, which is a key challenge for their application and functional reliability. Due to high surface energy, AgNPs have a natural tendency to agglomerate, which can diminish their unique catalytic and antimicrobial properties by reducing the available surface area [230]. The stability of AgNPs in a suspension is commonly quantified using zeta potential measurements, which indicate the magnitude of the electrostatic repulsion between adjacent, similarly charged particles. A higher absolute zeta potential value, typically above ±30 mV, is indicative of a stable colloidal system, as the repulsive forces are strong enough to overcome the van der Waals attractive forces that lead to agglomeration [231]. Concurrently, the Polydispersity Index (PDI) is used to assess the uniformity of the nanoparticle population, with lower PDI values (ideally < 0.3) signifying a narrow size distribution and reduced likelihood of Ostwald ripening, a process where larger particles grow at the expense of smaller ones, thus affecting shelf life [232]. To achieve such stability, capping or stabilizing agents are employed to coat the nanoparticles. These agents, which can include polymers like PVP, polysaccharides such as chitosan, or citrate, provide electrostatic or steric repulsion between particles, thereby preventing them from clumping together and ensuring their stability in colloidal suspensions [233]. The choice of capping agent is crucial as it influences not only the stability but also the size, shape, and biological activity of the AgNPs [234]. For instance, polyvinyl alcohol (PVA) has been shown to produce highly stable and small AgNPs with enhanced antibacterial activity [235]. The shelf life of AgNPs is also a significant consideration, as factors like oxidation, light exposure, and temperature can lead to degradation or re-nucleation over time, affecting their performance [26]. When these stabilized AgNPs are incorporated into polymeric nanofibers, the polymer matrix itself provides an additional layer of stabilization, physically entrapping the nanoparticles and preventing their migration and agglomeration, thereby ensuring a controlled and sustained release of silver ions and preserving the composite’s antimicrobial activity over time [236].

## 10. Applications of AgNPs

**Figure 4:**
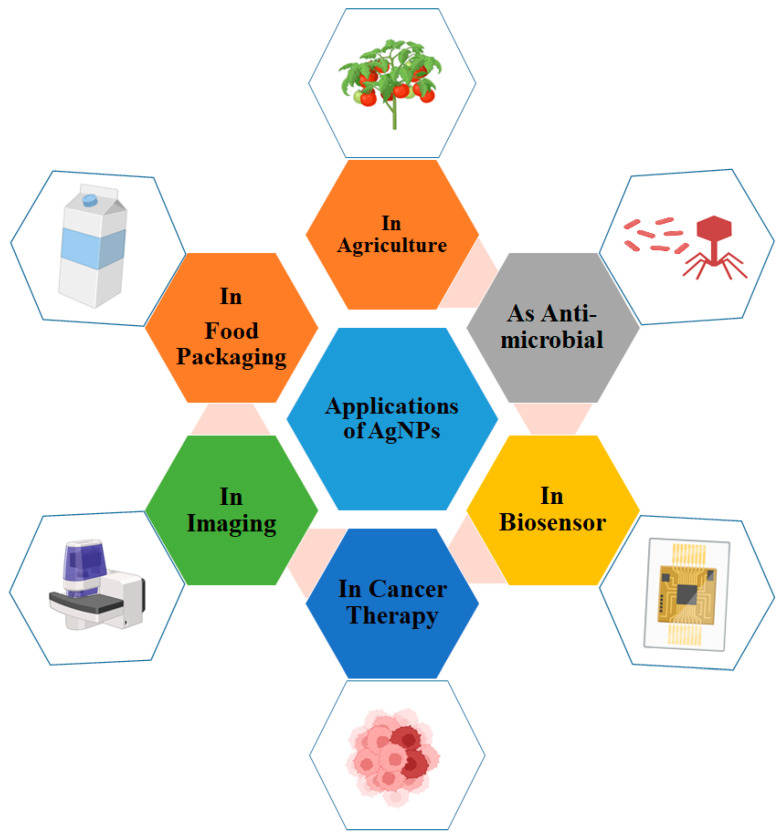
Applications of silver nanoparticles in different aspects. Adopted from [238].

AgNPs are one of the most attractive materials for industrial applications. They have been widely used as antibacterial agents; electronic products in the electronic industries; textile coatings; food storage; and many other features show their potential in environmental applications [14,70,237]. AgNPs provide a broad range of potential uses due to their unique characteristics, including as antimicrobial, antiparasitic, and antifouling agents; as agents for site-specific medication, water purification systems [95,181], mosquito control, agriculture, food safety, and food packaging [20]; and so many other applications (Figure 4) [13]. AgNPs have also been broadly used as antibacterial coatings for therapeutic applications, such as cardiovascular implants, wound dressings, catheters, orthopaedic implants, dental composites, nanobiosensing, and agriculture engineering [130,238]. Furthermore, AgNPs were used in the medical, plastics and textile industries [56]. Although AgNPs are used in a variety of applications, such as conductors [239], thin films [240], batteries [241], energy harvesting [242], and surface coatings [243], medical applications have received the most attention due to the rise of life-threatening diseases worldwide and multidrug resistance challenges in non-specific drug delivery [98,238].

## 11. Mechanism of Antimicrobial Activity of AgNPs

Microorganisms are potent and known to develop resistance to various antibiotics. However, as a result of the recent increase in bacterial resistance to various antibiotics, alternative therapeutic agents that are non-toxic to humans but toxic to microorganisms are urgently needed. Therefore, there is a strong case for the development of nanoparticle-mediated antimicrobial agents [13]. AgNPs are highly toxic to broad ranges of both Gram-negative and Gram-positive bacteria [14,244,245]. The antibacterial action of AgNPs on Gram-negative and Gram-positive bacterial strains is not the same but competes one over the other [76,181].

**Figure 5:**
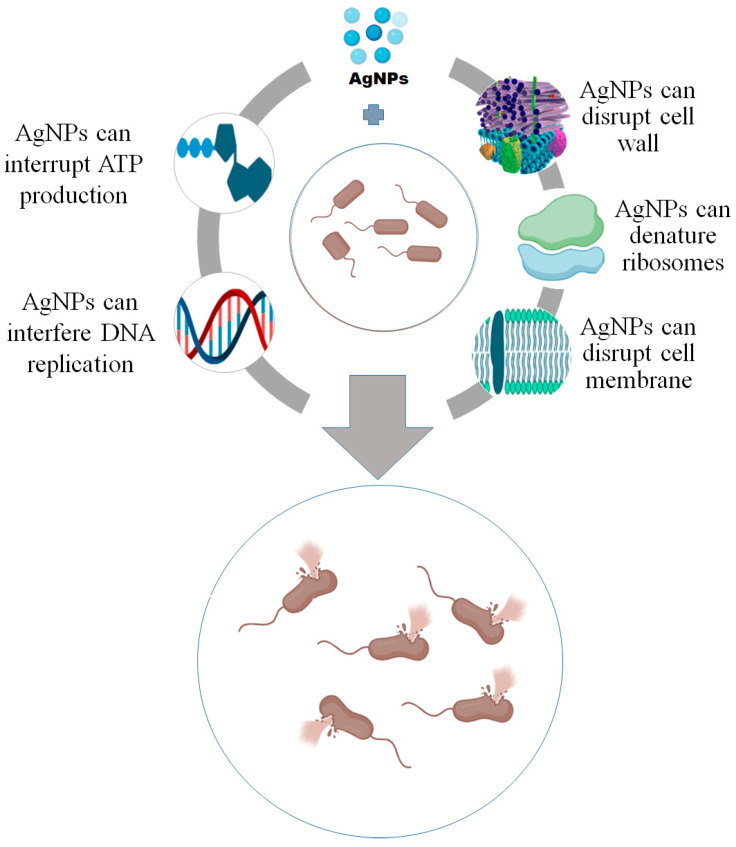
The antibacterial actions of AgNPs. (1) Disruption of cell wall and cytoplasmic membrane: silver ions (Ag^+^) released by AgNPs adhere to or pass through cell wall and cytoplasmic membrane. (2) Denaturation of ribosomes: silver ions denature ribosomes and inhibit protein synthesis. (3) Interruption of adenosine triphosphate (ATP) production: ATP production is terminated because silver ions deactivate respiratory enzyme on cytoplasmic membrane. (4) Membrane disruption by reactive oxygen species: reactive oxygen species produced by the broken electron transport chain can cause membrane disruption. (5) Interference of deoxyribonucleic acid (DNA) replication: silver and reactive oxygen species bind to DNA and prevent its replication and cell multiplication. (6) Denaturation of membrane: AgNPs accumulate in the pits of cell wall and cause membrane denaturation. (7) Perforation of membrane: AgNPs directly move across cytoplasmic membrane, which can release organelles from cell. Adopted from [247].

The antimicrobial activity of AgNPs can be attributed to the fact that smaller particles have a greater surface area available for interaction and will have more bactericidal effects than the larger particles [26,118,246]. Several mechanisms have been described for the antimicrobial activity of AgNPs (Figure 5) [247]. The interaction of nanoparticles with microorganisms begins with the adhesion of nanoparticles to the microbial cell wall and membrane. This adhesion is based on the electrostatic attraction between the negatively charged microbial cell membrane and the positively or less negatively charged nanoparticles [248,249,250]. After adhesion, morphological changes in the membrane structure are induced by the nanoparticles, thereby resulting in disruption of membrane permeability and respiratory functions through membrane depolarization, and ultimately disruption of the cell structure and cell death [250,251]. Moreover, nanoparticles have been reported to cause irregular pit formations on the cell wall, which further help the nanoparticles to enter into the periplasmic space and finally inside the cell [26,252].

After the interaction of nanoparticles with bacterial cells, peripheral damages and dense cavities on the cell surface can be observed [14,253,254]. AgNPs toxicity is also induced by the formation of free radicals, such as reactive oxygen species (ROS) [26,76,255]. The ROS include superoxide (O_2_^¯^), hydroxyl (^•^OH), hypochlorous acid (HOCl), peroxy (RCOO^•^), and hydrogen peroxide (H_2_O_2_). AgNPs promote the production of ROS, which can cause membrane disruption and disturb the permeability [36,98]. These free radicals damage the cell wall and biomolecules such as enzymes, proteins, lipids, and DNA [248,256]. ROS, or oxidative stress, forms and causes bacterial death [257]. AgNPs can disrupt proteins and phosphorus in the bacterial cell and destabilize ribosomal and DNA functions [258,259]. AgNPs are capable of interfering with the respiratory chain and cell division, which resulting in cell death [40,259,260]. It was also found that Ag^+^ ions produced from the AgNPs react with phosphorus, resulting in the stopping of DNA replication, or react with proteins containing sulfur, inhibiting the enzymatic functions [76,261]. Moreover, the binding of Ag^+^ ions and the proteins containing sulfur causes the breakdown of the bacterial cell walls and disturbs the mechanism of protein synthesis [98,100].

The integration of AgNPs into polymer matrices significantly influences their antimicrobial activity and can be tailored for specific applications. Polymer coatings, such as those made from polyvinyl alcohol (PVA) or polyurethane (PU), can enhance the stability of AgNPs by preventing their aggregation and improving their adhesion to surfaces [262]. This allows for a controlled and sustained release of silver ions, providing long-term antimicrobial action [36]. Encapsulating AgNPs within a polymer network can also reduce their cytotoxicity to human cells by limiting direct exposure while still allowing for the slow release of antimicrobial silver ions [36]. The choice of polymer and the method of integration can be optimized to control the release rate of silver ions and the overall effectiveness of the antimicrobial material [263]. For instance, hydrophilic polymers can facilitate the generation of silver ions, which is crucial for the antimicrobial activity of AgNPs in dry environment [262]. Furthermore, the surface charge of the polymer can influence the interaction with microbial membranes, with some studies showing that certain polymer coatings can enhance the bactericidal effect [264].

## 12. Polymeric Nanofiber–Nanosilver Composites

A composite is a material consisting of two or more different solid phases with distinguishable chemical, physical, structural, or biological properties [265]. As a result, a new class of only modified materials was created. The excess component in the form of a dispersion medium is normally known as a matrix. However, the other component, which is in the form of a scattered phase, is filler. Nanocomposites are formed when the fillers are either formed in situ or supplied ex situ with a particle size ranging between 1 and 100 nm along at least one dimension [266,267]. Nanocomposites are multiphase materials in which one phase contains nanoscale additives [266,268]. They are expected to show unusual properties resulting from the combination of each component. It has been classified into polymer matrix nanocomposites, polymer-layered silicate nanocomposites, ceramic-polymer nanocomposites, inorganic–organic polymer nanocomposites, and inorganic–organic hybrid polymer nanocomposites [54,269,270]. Polymer matrix nanocomposites are materials in which inorganic nanoparticles ranging in size from 10 to 100 nm must be dispersed in a polymer matrix [54]. Polymer nanoparticles have attracted the attention of researchers. Both in bulk and in solution, the materials offer unique thermal, optical, mechanical, and electrical properties. This significant improvement in the properties is due to the presence of nanoparticles and, their interaction with polymers, and the dispersion status [36,271]. Polymers, which are frequently used in medicine and the packaging industry, seem to be the most suitable matrices [33].

**Figure 6:**
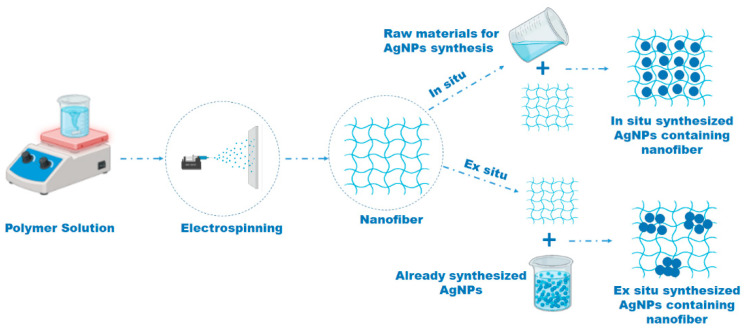
Differences in the in situ and ex situ preparation methods of AgNPs in addition to the difference in their aggregation.

Although nanoparticles have limited applications, those applications will dramatically expand if they are embedded in a matrix [33,272]. The incorporation of inorganic or organic materials improves and combines the properties of both phases in the final product [273]. Incorporating nanoparticles in polymer nanofibers has attracted considerable attention due to the possibilities of fabricating suitable materials for applications as catalysts, drug deliveries, medical gauzes, sensors, and gas separation or filtration nets [250,274], and antibacterial electrospun mats [275]. Nanoparticles can be incorporated into the polymer matrix in a variety of ways. In general, they can be divided into two main groups: in situ [20,276,277] and ex situ [278,279]. The manner in which the nanoparticles were inserted into the polymer matrix differs between these two techniques; in the in situ techniques, nanoparticles are generated directly in the polymer matrix from the entering components that react together. While producing polymer composites by ex situ techniques, prefabricated nanoparticles with defined size, shape, and quantity are introduced to the prepared polymer matrix [33]. Figure 6 is illustrating the differences in the in situ and ex situ preparation methods of AgNPs in addition to the difference in their aggregation.

AgNPs are used in pristine form or in combination with other materials for a variety of reasons, particularly in the biomedical field, with the advancement in the field of nanotechnology in many engineered products such as clothing, respirators, water filters, soaps, contraceptives, antibacterial sprays, and detergent, as well as in numerous household products [280]. Silver-containing nanofibers can be used as films, coatings, and fibers in electronics, sensors, filters, catalytic materials, water treatment, and food packaging due to the merits of both advantages of the antibacterial and optical enhancing properties of AgNPs and the high surface area and surface energy of nanofibers [34,281]. These composites containing AgNPs with multifunctional properties have a high scope for use as antimicrobial agents [250]. Moreover, they can also be used in the targeted delivery of drugs at a specific site [282,283].

## 13. Nanofibers

Nanofibers are ultra-fine solid fibers notable for their very small diameters (lower than 100 nm) [284], their large surface area per unit of mass, and their small pore size [285,286]. Nanofibers can be considered nanostructures, and this category of nanomaterials includes nanotubes as well as nanorods. Furthermore, nanofibers can contain nanoparticles in their bulk or on their surface, forming so-called nanofibrous composite materials [38]. Nanofibers, surface-functionalized with AgNPs, have attracted attention as an effective antimicrobial material [287,288,289]. Nanofibers produced from synthetic and natural polymers have drawn more attention among the diverse nanostructures that have recently been developed for use in practical applications, due to their ease of fabrication and the ability to control their compositional, structural, and functional properties [290,291,292,293]. In addition, due to numerous environmental concerns, current research focuses on the use of biodegradable polymers to develop various biomedical applications and components for environmental protection [294,295,296]. Currently, biodegradable and biocompatible polymers, such as collagen, alginate, chitosan, cellulose acetate (CA), gelatin, polyethylene glycol (PEG), polylactic acid (PLA), poly(glycolic acid) (PGA), poly(D, L-lactide-co-glycolide) (PLGA), polycaprolactone (PCL), polybutylene succinate (PBS), poly(vinyl alcohol) (PVA), poly(ethylene oxide) (PEO), and copolymers are used extensively to fabricate nanofibers [38,292,297,298].

## 14. Polymer Nanocomposites

Nanoscience and nanotechnology offer unique opportunities to create innovative combinations of nanoscale fillers and polymeric materials to obtain polymer nanocomposites with interesting properties [269,299]. Recent studies have further explored the incorporation of nanoparticles like Ag, ZnO, and TiO_2_ into PVA nanofibers, enhancing their antibacterial, mechanical, and thermal properties [269].

The choice of the polymer is an important point for obtaining fibers that can meet the demands of each application [272]. Usually, synthetic polymers exhibit reproducible behavior during electrospinning and provide highly uniform nanofiber mats [43]. Hydrophobic synthetic polymers allow for the preparation of mats that maintain their structure upon contact with aqueous media, and thus they have been extensively tested for liquid filtration [272,292,300]. Biopolymers, or naturally occurring polymers, have the advantages of being digestible or bioerodible, more biocompatible, and less immunogenic than synthetic polymers [43,301]. Many biopolymers are under investigation for production of electrospun mats containing AgNPs because of the antimicrobial activity in medical and healthcare nanofibers [36,302].

PVA is one of the most suitable polymers [303] that can be easily combined with natural polymers and crosslinked to make hydrogels since it is a semi-crystalline, biocompatible, non-toxic, hydrophilic polymer that also has excellent chemical and thermal stability and remarkable electrospinnability [304,305]. PVA is a polyhydroxy polymer that is water-soluble and has good fiber forming ability, biocompatibility, chemical resistance, and biodegradability [275,298,306,307]. Several researchers have reported electrospinning of PVA solutions [302,308], and PVA has been reported in numerous publications to be a good choice for preparing colloidal suspensions due to its significant advantages such as processability and high transmittance [309,310].

In order to enhance the functionality of the nanofibrous scaffolds, PVA is often combined with cyclodextrins (CDs) [311]. CDs are a family of cyclic oligosaccharides with a cyclic molecular structure, and are composed of cyclic α (1, 4) linked pyridoglycan units. The three most common natural CDs contain 6, 7, or 8 units of glucopyranose in the cycle and are termed α-CD, β-CD, and γ-CD, respectively [42,44,312,313]. Although they are not polymers, CDs have attractive features when incorporated into electrospun fibers [43]. The CDs are considered one of the pioneers of super molecules with broad applications in various fields, such as host–guest interaction, molecular recognition, drug delivery, enzyme catalysis, foods, cosmetics, pharmaceuticals, home/personal care, textiles, and pesticides [44,314]. The functionalization of nanofibers with cyclodextrins will provide unique properties for traditional composite fibers. For instance, nanofibers can be used for filtering tiny particles as well as acting as barriers for liquid/vapor penetration due to the large surface area of their nanoporous structure [315,316]. Furthermore, the hydrophobic cavity of CDs enables them to form host–guest complexes with different small molecules and macromolecules [312]. Among these CDs, β-cyclodextrin (β-CD), composed of seven glucose units with a moderately truncated cone-shaped hole, is commercially available and has been widely used in industry [317]. β-CD possess a toroid structure with a lipophilic inner surface and a hydrophilic outer surface that make them efficient materials for the formation of non-covalent host–guest inclusion complexes with a wide range of organic and inorganic molecules [42,318,319]. The addition of β-CD in polymer nanofibers would be extremely attractive since composites have a novel feature that can potentially improve the application areas of nanofibers [7]. Many researchers have attempted to modulate the function of polymeric scaffolds by combining various types of polymers. Dos Santos et al. [320] reported that CDs enhanced stability during the spinning process of PVA/β-CD. The β-CD functionalized nanofibers and their assemblies have potential applications in biomedical fields due to their high aqueous solubility, low toxicity, and fewer side effects [311,321]. Furthermore, β-CD easily reacts with cross-linking agents to give hyper-cross-linked polymers that are water-insoluble [318]. Recent studies have also explored the preparation and characterization of β-cyclodextrin grafted chitosan nanofibers via electrospinning for dye removal, indicating the expanding applications of CD-functionalized nanofibers [322]

Polyethylene oxide (PEO) is a crystalline, thermoplastic polymer. Unlike many other polymer systems, PEO is commercially available in a wide range of molecular weights. PEO is colorless, odorless, and stable against heat and hydrolysis, acts as an inert substance for many chemical reagents, and is a non-toxic polymer. PEO is an excellent candidate for biomedical applications because of its biocompatibility, non-immunogenicity, and interesting physicochemical characteristics [323]. It is particularly appropriate for use as scaffolds in tissue engineering [324,325,326] and biocompatible coatings [327,328], as well as other wide application fields, such as filtration for CO2 separations [329], and energy storage applications [330]. PEO, in small amounts, acts as a plasticizer in electrospinning processes [324,331]. Fortunately, even the negligible presence of PEO (up to 2%) in the solutions of the materials that, in their pure forms cannot be electrospun, such as chitosan, keratin, and other protein-based materials, completely changes their disposition to be electrospun [332].

To combine the advantages of various polymers and overcome their limitations, the desired properties were obtained by using mixtures of these polymers instead of single polymers by optimizing the ratio between the components of the mixture [333]. By combining the advantages of PVA, PEO, and β-CD, it is expected that PVA/PEO/β-CD hybrid scaffolds will have strong mechanical features, biocompatibility, and biodegradability properties. This will offer more desirable properties and functionality for applications in various fields. Thus, the properties of electrospun scaffolds can be tailored with desired new functions by selecting a combination of appropriate components and adjusting the component ratio. In addition to taking advantage of the material’s compositions, the fabrication process, through which the fiber diameter, morphology, and scaffold porosity can be manipulated, also plays an important role on the properties and functionality of the scaffolds [334]. PVA, PEO, and β-CD are well known as non-toxic, biodegradable, and water-soluble materials [317,335,336]. These features are essential for many applications, such as nanoagriculture, tissue engineering, and other biomedical applications [337]. The slow biodegradation rate of PEO compared to PVA and PVA/PEO/β-CD blends makes it the preferable polymer to utilize in applications where the release rate of active materials, such as drugs, fertilizers [338], and active metal NPs embedded in the nanofibers, must be controlled for long time periods.

Nanofiber membranes are prepared by the electrospinning process of polymers, and the application possibilities are limited to fiber scaffolds [339]. The production of antimicrobial nanofibers generally follows the strategy of incorporating biocide into the fibers. This can be achieved by uniformly mixing the active agent in the polymer solution before electrospinning, entrapping the active agent in the fiber core by coaxial electrospinning, entrapping the active agent in nanostructures before dispersing it in the electrospinning solution, or attaching the active agent to the surface of the fiber. Until now, various active substances, including antibiotics, biocides, metallic nanoparticles, metal oxide nanoparticles, and natural bioactive compounds, have been used [38]. Thus, various functional nanofiber membranes have been prepared by electrospinning in combination with multifunctional materials, among which nanofiber–nanoparticle hybrids show great potential to combine the advantages of nanoparticles with the properties of polymers [333]. For instance, functional composite nanofibers can be produced by incorporation of metal nanoparticles, such as silver or gold, into electrospun polymeric nanofibers [340,341,342]. AgNPs have attracted considerable attention due to their unique optical, electronic, catalytic and antimicrobial properties [259,343,344]. AgNPs/polymer composites, which function as bactericides, have been applied to complicated cases of infected burns, purulent wounds, and wound healing matrix [14,345]. Several studies aimed at incorporation of AgNPs within electrospun nanofibers which can enable production of functional nanofibrous composites by combining the unique properties of nanofibers with that of AgNPs [321,346,347]. Yang et al. [274] first prepared ultrafine polyacrylonitrile (PAN) fibers containing AgNPs via electrospinning. Andrade et al. [313] reported that β-CD-coated AgNPs reduced more than 99% *E. coli* as compared to pristine β-CD. Celebioglu et al. [348] developed an electrospun AgNP/β-CD nanofiber composite membrane, which showed good antibacterial activity against Escherichia coli and Staphylococcus aureus. Also, Celebioglu et al. [321] reported that the electrospun PVA/hydroxypropyl-β-cyclodextrin (HPβ-CD) nanofibers incorporating AgNPs were successfully electrospun. Furthermore, the synthesis of nanofibers composed of AgNPs and PVA has attracted increasing attention due to their unique physical and chemical properties. The AgNP/PVA hybrid nanofibers show various applications as sensors, antimicrobial materials, wound dressings, catalytic agents, and dye-sensitized solar cells [349]. For all of the above reasons, PVA containing AgNPs is appropriate for the application of antimicrobial benefits, so that the method for synthesis of PVA loaded with AgNPs is under consideration by scientists [345]. Hong, [350] engineered PVA-based mats containing AgNPs by means of a precursory solution of the corresponding polymer and AgNO3 with a subsequent thermal or ultraviolet (UV) posttreatment. The PVA-based mats produced exhibited excellent antibacterial activity against Staphylococcus aureus and Klebsiella pneumoniae. Although the water solubility of PVA might be a drawback when using it as a wound dressing, this can be overcome by simple thermal treatment without having a negative effect on the final properties. Li et al. [351] also reported that electrospun composite nanofibers comprised of PVA/chitosan/AgNPs can effectively be used as a wound dressing agent due to its proven effectiveness in inhibiting bacterial growth. Other research related to the fabrication of nanofibers in the presence of AgNPs has been reported. For example, He et al. [41] successfully developed feather keratin/poly (vinyl alcohol)/poly (ethylene oxide) (FK/PVA/PEO) composite nanofibers using the electrospinning process. These nanofibers exhibited antibacterial activities against both Gram-positive (*S. aureus*) and Gram-negative (*E. coli*) bacteria. Nguyen et al. [345] synthesized AgNP/PVA nanofibers and tested their antibacterial activity toward Gram-positive bacteria, Staphylococcus aureus and Gram-negative bacteria, Escherichia coli. Furthermore, Hamza et al. [352] successfully fabricated AgNP/PVA/zinc oxide (ZnO) nanofiber mats using the electrospinning technique. They also found that the AgNP/PVA/ZnO nanofibers exhibited better antibacterial efficiency compared to the PVA nanofibers. Therefore, electrospun composite nanofiber scaffolds functionalized with AgNPs are considered an important asset in future medicine [280]. Also, George et al. [347] successfully fabricated casein/polyvinyl alcohol (CAN/PVA) films and nanocomposites functionalized with AgNPs using a solution casting technique. The nanofibers and films were functionalized by incorporating AgNPs to formulate smart materials for enhanced non-linear applications.

Nanofibers can be prepared using various techniques, including electrospinning, drawing, template synthesis, phase separation, and self-assembly [14]. The electrospinning method was found to be one of the most efficient, straightforward, and versatile due to its relatively simple and cost-effective setup [292,342,343,353].

## 15. Electrospinning

**Figure 7:**
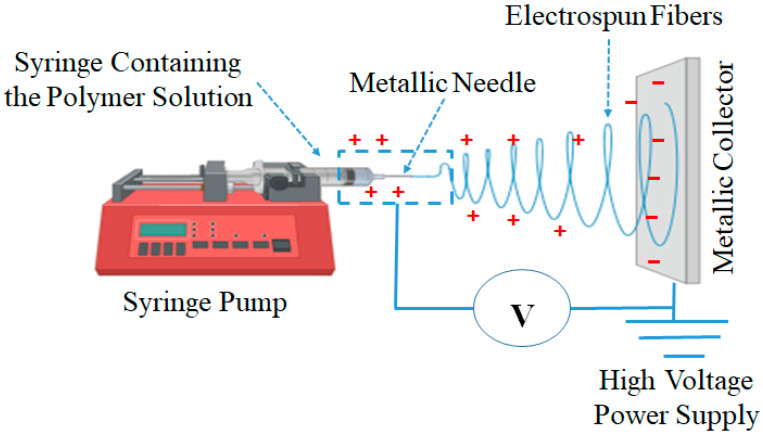
Scheme of the electrospinning setup and process. Adopted from [295].

Since the 1930s, electrospinning has been applied to produce various nanofibrous fabrics for various applications including filters, semi-permeable membranes, skin masks, clothing, and medical materials [354]. So far, nanofibrous scaffolds have been fabricated using phase separation, self-assembly, and electrospinning [355]. Among them, the electrospinning process has attracted considerable interest since it produces nanofibrous scaffolds with high porosity and an adjustable pore size distribution [356]. Electrospinning is a versatile technology to produce polymer nanofibers from solutions or melts using electrostatic forces (Figure 7) [286,295,357]. The electrospinning process is simple, cheap, and easy. Therefore, it has been used as an efficient method to produce ultra-fine nanofibers [61,358]. These nanofibers have excellent properties such as an extra-large surface to volume ratio, small fiber diameters, a porous surface, and a more functional surface [296,359]. Therefore, they can be applied in several fields, such as filters, wound dressing, protective clothes, medical fields, sensors, drug delivery, tissue engineering, cosmetics, and biomedical fields [61,360,361,362,363,364].

A polymer solution is electrospun into a continuous filament under a high-voltage electric field. Then these filaments are deposited on the collector [36]. This process depends on several factors, such as viscosity, surface tension, and solution conductivity [342,365,366]. Electrospinning enables the efficient fabrication of fibers with diameters ranging from a few nanometers to several micrometers. The electrospun nanofiber mats have a high surface area per unit mass, high porosity, and high gas permeability. Due to the listed characteristics, nanofibers have found numerous applications in different areas of daily life [38,286].

## 16. Application of Polymeric AgNP–Nanofiber Composites

Polymer matrix-based nanocomposites attract attention in various research fields because of their numerous and vast applications, such as in construction and infrastructural materials, biomedical fields, aerospace and automobile parts, textile industries, etc. [266,269]. They can be found in everything, from tiny objects like chopsticks to larger ones like trash bins, or from fundamental structural components to well-established technological equipment. All this is due to the diversity of their characteristics [266]. Many electrospun antimicrobial fibers are based on metal or metal oxide nanoparticles (such as silver, copper, ZnO, or TiO_2_) that have been utilized on a macroscale for centuries [38,367]. These nanofibers are an example of new delivery systems based on the reservoir concept, in which the polymer structure surrounds the reservoir with a release rate governed by the rate of polymer degradation, the rate of diffusion, or the detachment of the surface coating [368]. With the merits of the AgNPs’ properties and the high surface-to-volume ratio of nanofibers, the application of the AgNPs–nanofibers has developed significantly [34]. The most well-known antimicrobial nanostructures are AgNPs, which exhibit a broad spectrum of antimicrobial activity [36,367,369] and a rare incidence of resistance [370]. Due to the unique antimicrobial bioactivity of AgNPs, AgNP–nanofiber structures can be widely used in many applications, which becomes important especially in the biomedical industry [36,210].

### 16.1. Antimicrobial Materials

Polymer-based metallic nanoparticles are widely explored for their versatility, biodegradability, eco-friendliness, and nature biocompatibility [371]. Recently, with the misuse of antibiotics, different types of drug-resistant bacteria emerged [372], and the combination of various types of polymer-based metallic nanoparticles can also generate excellent antibacterial capability against drug-resistant bacteria [333]. The accumulation of AgNPs in mitochondria results in mitochondrial dysfunction. In addition, AgNPs disrupt the DNA structure, leading to non-replication and effective suppression of bacterial multiplication. These two fundamental factors lead AgNPs to possess a powerful bactericidal potential [34]. Many items such as textiles, keyboards, and medical devices now contain AgNPs that continuously release small amounts of Ag^+^ to provide antimicrobial protection [373]. AgNPs have also been used to develop various bioactive materials, including polymer composites, due to their high antimicrobial activity [17]. Antimicrobial activity studies were typically performed using diffusion techniques, and showed that AgNP-loaded nanofibers exhibited excellent antimicrobial properties against both Gram-positive and Gram-negative bacteria [35,36,38,374].

The antimicrobial activity of AgNPs is dependent on the nanomaterial’s surface area [375]. The highest concentrations of released Ag^+^ ions were observed from AgNPs with the largest surface area. In contrast, the low release of Ag^+^ ions was found for AgNPs with small surface area, resulting in weak antimicrobial properties [376]. Most electrospun nanofibers have no effect on the reproduction of microbial cells by their own, but only in the presence of AgNPs [34]. For instance, according to Shi et al. [377] pure nylon 6 nanofiber has no antibacterial activity, but when AgNPs are incorporated into the polymer matrix, it exhibits a 99.9% inhibition to *E. coli* at a silver precursor concentration of 0.5 weight% and a 99.999% inhibition at a concentration of 1.25 weight%. These characteristics make the silver-containing nanofibers an excellent candidate for biotextiles and wound dressings [34]. Also, Rzayev et al. [378] reported that poly (vinyl alcohol-co-vinyl acetate)/octadecyl amine-montmorillonite) (P(VA-co-VAc)/ODA-MMT) nanofibers loaded with AgNPs showed strong antimicrobial activity against fungi (*Candida albicans*, *tropicalis*, *glabrata*, *keyfr*, and *krusei*) and bacteria (*S. aureus* and *E. coli*).

Although, there is a lot of debate about which molecule is responsible for causing microbial cell kill—is it the Ag^+^ or the AgNPs?—many authors attribute the primary antimicrobial activity to the dissolved Ag^+^ released by AgNPs, as the smaller particles are dissolved faster, creating higher numbers of silver ions near the microbial cells, which enhances the killing process [379]. However, others show that direct contact with AgNPs can damage the membranes, generate ROS at the particle surface, and disrupt the formed biofilm. All these actions were reported for AgNPs even when the silver ions were controlled or matched. Recent comparative studies have concluded that both released Ag^+^ and particle-specific interactions play a role [380,381,382]. On the other hand, some researchers reported multiple risks from these nanoparticles, including ROS-mediated injury in mammalian and endothelial cells, lysosomal and mitochondrial damage, and autography disruption; however, the magnitude varies according to the size and the coatings of the nanoparticles [383,384]. However, regarding food-contact contexts, the scientists of the FDA reported that the reducing ingredients in foods can generate and transform the silver particles at the interface, and will potentially alter the exposure of consumers compared with neat lab media [385]. For these reasons of risk potential, polymer matrices, coatings of AgNPs, and storage conditions can be adapted to limit the migration of the nanoparticles while preserving the antimicrobial surfaces. Both the EFSA and the FDA highlighted in their interval guidance and reassessments that the key uncertainties demand standardized ion-release testing in complex foods, determination of the long-term storage effects on dissolution, and regular hazard assessment of the harmonized dose metrics [386].

### 16.2. Wound Dressings

In comparison to cotton gauze dressings, metallic nanocomposite scaffolds made from polymer matrices are found to be more advantageous. Cotton dressings are known as passive dressings since they only served as a structural framework material to cover the infected wounds [266]. But recently, bioactive and interactive dressing materials in the form of films, hydrogels, and sponges are being identified to benefit the process of wound healing [387]. AgNPs have a wide antibacterial spectrum covering aerobic, anaerobic, and Gram amphoteric bacteria [388], a low incidence of resistance [389], and persistent antibacterial activity [390], so they have been frequently used in antibacterial wound dressings [12,34,391]. Abdelgawad et al. [392] reported that the PVA/chitosan–AgNP nanomaterial mats have been developed for antimicrobial applications. The PVA/CS-AgNP mats have shown good antibacterial activity against *E. coli*, which promotes good wound dressing material. In addition, according to Anisha et al. [393] AgNP/chitosan–hyaluronic acid (sponge) was synthesized as a dressing material against diabetic foot ulcers. The antibacterial activity was tested against *P. aeruginosa*, *E. coli*, *K. pneumoniae*, *S. aureus*, and methicillin-resistant *Staphylococcus aureus* (MRSA). The results obtained have suggested the applicability of composite sponges as a dressing material for diabetic foot ulcers and infected wounds. Furthermore, the researchers compared the antibacterial properties of silver-containing nanofibers with other polymers commonly used for wound healing without the presence of AgNPs. The results show that PVP/CNC/Ag nanofiber composites showed improved antibacterial activity against both *S. aureus* and *E. coli* than the PVP/CNC nanofiber composite [394].

### 16.3. Food Packaging Materials

Polymer-based metallic nanoparticles used for food packaging applications can be natural biopolymers with inorganic metal/metal oxide nanoparticles. Metal/metal oxide nanoparticles of silver, gold, copper, and zinc usually impart antimicrobial property and are utilized to develop antimicrobial food packaging products [14,266]. In order to delay food spoilage or bacterial contamination, AgNP-loaded electrospun membranes with antibacterial properties have been tested in food packaging materials [390,395]. For instance, Chaudhary et al. [395] used an electrospun AgNP/PAN composite filter media to cover a nutrient media under room conditions and allow ambient air to pass through the filter media. After two months, the nutrient media protected by the nanofibrous filter maintained free of bacterial growth, while the unprotected nutrient media exhibited microbial growth. Also, Castro-Mayorga et al. [396] reported electrohydrodynamic processing to create a multilayer system consisting of a poly (hydroxy alkanoate) (PHA) substrate and an electrospun PHA coating containing AgNPs, taking advantage of the AgNP-loaded electrospun nanofibers that could prevent microbial outbreaks in food packages and food contact surfaces. The produced materials reduced the *Salmonella enterica* population below the detection limits at a very low silver loading of 0.002% wt.

### 16.4. Antimicrobial Nanopaints

The applications of nanomaterials with antimicrobial properties are not limited to therapeutic applications but are also useful for the development of antimicrobial products such as coatings and water filters [397]. It is profitable to combine AgNPs with polymer matrix to form multifunctional composite coatings, which could be used in antimicrobial applications [398]. In the application of AgNP/polymer nanopaints, AgNPs are produced in a polymer solution using a one-step method, followed by a drying process. The resulting drying oil containing AgNPs is a good coating material that can be applied to different surfaces, including glass, wood, and polystyrene [399]. The surface covered with the nanopaints shows excellent antibacterial properties by killing both Gram-negative and Gram-positive pathogenic bacteria [399]. For instance, nanosilver-based wall paint would prevent the growing of mold inside buildings and the growth of algae on outside walls [400].

### 16.5. Water Filtration and Treatment

Among the various types of nanosized antimicrobial materials, namely ZnO, CuO, and TiO_2_, the AgNPs have been reported as the most effective antimicrobial agents [21,275,401,402]. When paired with hydrogen-bonded multilayers assembled on magnetic microspheres, it can be delivered and located in a specific region without contaminating the environment by using magnetic fields [403]. A bifunctional Fe_3_O_4_ and AgNPs composite with superparamagnetic and antibacterial properties has been prepared and proven to have excellent antibacterial ability against *E. coli*, *S. epidermis*, and *B. subtilis* [404]. In addition to the advantages of supermagnetism, which allows easy removal of the materials from water, mesoporous polymer nanofiber membranes can be designed with specific pore sizes and desired filtration properties to enhance the efficiency of water treatment and their recyclability. The nanocomposites of supermagnetics/AgNPs/polymer nanofibers can be a promising water disinfectant [404,405].

### 16.6. Catalyst for Hydrolysis/Electrolysis of Polymer Matrix

Accelerating the hydrolysis of bulk material is another application for incorporating AgNPs into polymer nanofibers. During the fabrication of AgNP/polymer composites, AgNPs exhibit remarkable catalytic properties for the hydrolysis and electrolysis of organic materials [406,407]. Furthermore, the catalytic activity can be adapted by controlling the size of silver particles and the polymer matrix. In general, the smaller particle size has a higher catalytic activity [407] with a greater number of reaction sites per surface area [408]. This size dependence becomes even more important when the particle size is reduced at the nanoscale. According to studies, AgNPs were synthesized and subsequently incorporated in the polymer matrix of polyvinyl acetate (PVAc) for the preparation of the AgNP/PVAc nanocomposite [409]. By adding AgNPs to a polymer matrix, the hydrolysis of this hybrid material can be accelerated. As a result of their strong bond with the PVAc polymer chain, the AgNPs can continue to function as a catalyst even when the polymer chains’ backbones are broken during hydrolysis. AgNPs cause the hydrophobic PVAc polymer to become hydrophilic and dissolve in particular solvents, leading to accelerated hydrolysis.

## 17. Future Perspectives and Limitations

Despite a number of technical obstacles, future studies on green-synthesized silver nanoparticles (AgNPs) and AgNP-incorporated polymeric nanofibers are expected to advance toward standardization, scalability, and multifunctional integration. Regulatory acceptance and reproducibility are complicated by the synthesis side of plant- and microbe-mediated AgNP production, which is still plagued by batch-to-batch variability in phytochemical content, incomplete reporting of quantitative parameters (yield, polydispersity index, zeta potential), and a lack of mechanistic understanding of Ag^+^ release versus particle-driven effects. Better control over nanoparticle dispersion, aggregation prevention, and long-term stability under real-world conditions like humidity, UV exposure, and mechanical stress are necessary for both ex situ and in situ incorporation techniques for polymeric nanofibers. Real-world research paths include (1) creating omics-guided strain or plant selection for predictable capping chemistry; (2) incorporating real-time ion-release monitoring into antimicrobial performance tests; (3) developing solvent-minimized, scalable electrospinning and melt-processing routes that are compatible with green AgNPs; and (4) creating interfaces between polymers and nanoparticles that modulate Ag^+^ release rates to meet safety thresholds without sacrificing efficacy. The biggest technical challenges are harmonizing international regulatory standards for nano-enabled materials, migration testing under complex-use scenarios (such as food-contact, biomedical), and scalable process control for uniform size/composition. Multidisciplinary efforts integrating toxicology, green chemistry, nanomaterials engineering, and regulatory science into an iterative, application-specific development cycle will be necessary to address these.

## 18. Conclusions

Nanotechnology, especially the development and application of AgNPs, has emerged as a promising field in biological and nanomedical research. AgNPs exhibit significant antimicrobial properties, making them valuable in the design of advanced products, such as wound dressings, medical implants, and biosensors. Green-synthesized AgNPs and their polymeric nanofiber composites represent a major advancement in nanotechnology. Recent progress in the synthesis of AgNPs highlights the potential for more sustainable and eco-friendly production methods that avoid hazardous chemicals. Moreover, the incorporation of AgNPs into polymeric nanofiber composites offers a powerful strategy to enhance stability and allows the development of functional materials with enhanced antimicrobial activity, biodegradability, and non-toxicity, which are essential for various applications ranging from advanced wound dressings to water purification systems. The development of AgNP-based nanocomposites with tailored properties remains an exciting area of research, with significant implications for medicine and environmental protection. While AgNPs and their composites hold great promise for a wide range of applications, ongoing research is crucial to overcome existing limitations. By advancing the understanding of their biological interactions, optimizing production methods, and ensuring their safety, AgNP-based materials could become integral components in numerous products. Future research must address the urgent need of scalable and cost-effective green synthesis, as many current methods lack high-yield production suitable for industrial applications. Ensuring the long-term stability and shelf life of AgNP-based composites is another critical hurdle, since aggregation and degradation can reduce effectiveness over time. Most importantly, a deeper understanding of the potential nanotoxicity is required, including the environmental effects of silver ion leaching and the implications for human health—factors central to regulatory approval. Overcoming these challenges in synthesis, stability, and safety is crucial to unlocking the full transformative potential of AgNPs and their composites. Continued research in this field promises further innovations in sustainable and effective nanomaterials.

## Figures and Tables

**Table 1:** Summary of some recent studies on the green synthesis of AgNPs along with its quantification techniques and applications.

Green Source	Scientific Names	Size (nm)	Shape	Wavelength (nm)	Zeta Potential (mV)	Applications	Reference
plant	*Ligustrum lucidum*	13	Spherical, triangular, polygonal, irregular	438	-	A novel fungistat not only for comprehensive control of plant fungi	[160]
plant	tea	20–50	spherical	405–415	−24.6	Antibacterial activity	[161]
plant	*Lallemantia royleana* (Benth. in Wall.)	34.47 ± 1.6	spherical	425	−24.1	Biopharmaceuticals and catalytic applications	[162]
plant	*Allium cepa*	19.47 ± 1.12	spherical	439	−13.1	Biomedical activities and also has other possible industrial applications	[163]
plant	*Juglans* *regia*	80-90	spherical	420	−67.2	Photocatalytic degradation of effluent dye	[164]
plant	*Alpinia nigra*	49.36	Spherical	400–500	-	Antimicrobial activity	[165]
plant	*Berberis vulgaris*	30–70	Spherical	450	84.85	Antibacterial activity	[166]
plant	*Coleus forskohlii*	10–50	Trigonal, hexagonal, spherical, rod	420	-	Antimicrobial activity	[167]
Bacteria	*Leclercia acarboxylata*	17.43	spherical	423	-	Antimicrobial activity	[31]
Bacteria	*Bacillus brevis*	41–68	Spherical	420	-	Antibacterial activity	[168]
Bacteria	*Bacillus* sp.	22–41	Spherical	447	-	Antifungal activity	[169]
Bacteria	*Pseudoduganella* *eburnean*	8–24	Spherical	448	-	Antimicrobial activity	[170]
Fungus	*Setosphaeria* *rostrata*	2–20	Spherical	400	-	Antibacterial activity	[171]
Fungus	*Penicillium* *oxalicum*	60–80	Spherical	600	-	Antibacterial activity	[172]
Fungus	*Trichoderma asperellum*	15.5 ± 2.5	Spherical	410	-	Antifungal activity	[173]
Fungus	*Arthroderma fulvum*	15.5 ± 2.5	Spherical	420	-	Antifungal activity	[174]
Fungus	*Talaromyces purpureogenus*	30–60	Spherical	380–470	−19.6	Antibacterial and antioxidant activities	[175]
Algae	*Portieria hornemannii*	35–50	Spherical	418	−44.5	Skyscraping activity against fish pathogens	[176]
Algae	*Graesiella emersonii*	4–35.02	Spherical	400–415	±20–30	Antibacterial activity	[177]
Algae	*Enteromorpha compressa*	4–24	Spherical	421	-	Biomedical and pharmaceutical applications.	[178]

**Table 2:** Key techniques for the characterization of AgNPs.

Technique	Acronym	Information Provided	Principle
UV–Visible Spectroscopy	UV-Vis	Confirms the formation of AgNPs and provides preliminary data on size and stability.	Measures the absorption of light. AgNPs exhibit a unique SPR peak, typically between 400–450 nm.
Transmission Electron Microscopy	TEM	Determines particle size, size distribution, and morphology (shape).	An electron beam is transmitted through an ultra-thin sample, creating a high-resolution 2D projection image of the nanoparticles.
Scanning Electron Microscopy	SEM	Visualizes the surface morphology of AgNPs, especially when deposited on a substrate or embedded in nanofibers.	Scans the sample surface with a focused electron beam to produce images of the surface topography and composition.
Dynamic Light Scattering	DLS	Measures the hydrodynamic diameter (size in solution) and size distribution.	Analyzes the fluctuations in scattered light intensity caused by the Brownian motion of particles in a suspension.
Zeta Potential Analysis		Determines the surface charge and predicts the colloidal stability of the AgNPs suspension.	Measures the electrophoretic mobility of particles in an electric field. High absolute values (>±30 mV) indicate good stability.
X-ray Diffraction	XRD	Identifies the crystalline structure and phase purity of the AgNPs.	Measures the scattering of X-rays as they pass through a sample, producing a diffraction pattern characteristic of the material’s crystal lattice.
Fourier-Transform Infrared Spectroscopy	FTIR	Identifies the functional groups of capping agents on the nanoparticle surface.	Measures the absorption of infrared radiation by the sample, revealing the vibrational modes of chemical bonds present.
Energy-Dispersive X-ray Spectroscopy	EDS/EDX	Confirms the elemental composition and purity of the sample.	Analyzes the X-rays emitted from a sample bombarded by an electron beam to identify the elements present. Often coupled with SEM or TEM.

**Table 3:** Comparing some characteristic parameters of different methods used for the green production of silver nanoparticles.

Synthesis Method	Particle Size	Polydispersity/PDI	Zeta Potential (mV)	Scalability Challenges	Reference
Aqueous rhizome extract	TEM: ~5–40 nm (average < 20 nm)	NR	NR	Reproducibility and standardization of extract concentration; lack of absolute yield metrics—both limit direct scale-up planning	[223]
Aqueous leaf extract	TEM/XRD: ~15 ± 5 nm	Described as monodispersed	NR	Good size control in lab but process depends strongly on extract composition and kinetics (batch-to-batch variability); scale-up needs extract standardization and control of mixing/heat transfer.	[224]
Aqueous Geranium (Pelargonium) leaf extract	TEM/SEM: ~30–44 nm	narrow size distribution	−20 to −30 mV	Controlling extract phytochemical content at large scale is a major challenge.	[225]
Aqueous leaf extract	DLS/FESEM: ~28–32 nm	NR	−41.4 mV	Scaling requires consistent extract composition and filtration	[226]
Culture filtrate	DLS: 11–42 nm	Described as high dispersity	−26 ± 0.2 mV	Variability in microbial metabolite composition; absence of quantitative yield measurement; controlling consistency at scale	[227]
Extracellular secretions from fungi (Cladosporium, Penicillium, Purpureocillium)	TEM: mostly < 20 nm; DLS: Cladosporium/Purpureocillium ~166 nm; Penicillium ~124 nm	PDI: Cladosporium 0.074; Purpureocillium 0.279	Cladosporium −15.7 mV; Penicillium −17.8 mV; Purpureocillium −13.0 mV	Scaling extracellular fungal systems requires consistent enzyme/metabolite production; larger hydrodynamic sizes might affect functionality and processing	[228]
Fungal extract	DLS Z-average: 240.2 nm	PDI: 0.720 (high; polydisperse)	−19.5 mV	Very large, polydisperse particles with borderline zeta (moderate stability); significant challenges for reproducibility and downstream uniformity	[175]
Extracellular pigment from fungus	NR	Not detailed	−24.8 ± 7.2 mV	Downstream drying adds step; pigment variability; moderate zeta suggests reasonable stability but consistency still a hurdle	[229]

Legend: PDI = polydispersity index (hydrodynamic), NR = not reported.
